# *Mycobacterium lepromatosis* as Cause of Leprosy, Colombia

**DOI:** 10.3201/eid2805.212015

**Published:** 2022-05

**Authors:** Nora Cardona-Castro, María Victoria Escobar-Builes, Héctor Serrano-Coll, Linda B. Adams, Ramanuj Lahiri

**Affiliations:** Colombian Institute of Tropical Medicine–CES University, Antioquia, Colombia (N. Cardona-Castro, M.V. Escobar-Builes, H. Serrano-Coll);; US Department of Health and Human Services, Health Resources and Services Administration, Health Systems Bureau, National Hansen’s Disease Program, Baton Rouge, Louisiana, USA (L.B. Adams, R. Lahiri)

**Keywords:** *Mycobacterium lepromatosis*, *Mycobacterium leprae*, leprosy, Colombia, tuberculosis and other mycobacteria, bacteria, Hansen disease, *Suggested citation for this article*: Cardona-Castro N, Escobar-Builes MV, Serrano-Coll H, Adams LB, Lahiri R. *Mycobacterium lepromatosis* as cause of leprosy, Colombia. Emerg Infect Dis. 2022 May [*date cited*]. https://doi.org/10.3201/eid2805.212015

## Abstract

Leprosy is a granulomatous infection caused by infection with *Mycobacterium leprae* or *M. lepromatosis*. We evaluated skin biopsy and slit skin smear samples from 92 leprosy patients in Colombia by quantitative PCR. Five (5.4%) patients tested positive for *M. lepromatosis*, providing evidence of the presence of this pathogen in Colombia.

The primary causal agent of leprosy is *Mycobacterium leprae*; however, as of February 2012, *M. lepromatosis* has been established as another etiologic agent that is still underexplored in many leprosy-endemic countries (*1*). Dual infections caused by both species have also been reported (*2*). The similarities between these bacteria initially led researchers to think *M. lepromatosis* was a new strain of *M. leprae*; however, it is now considered a new species because of ≈9% difference in whole-genome sequences (*3*). 

The global prevalence and extent of *M. lepromatosis* infection are still unknown. Also unknown is whether *M. lepromatosis* can cause substantially different disease severity from *M. leprae* manifested as nerve damage, leprosy reactions (type I/II), relapse rate, and overall prognosis; these factors are essential to understanding the clinical implications and case management of patients with *M. lepromatosis* infection or co-infection. We report the presence of *M. lepromatosis* in patients in Colombia.

We performed *M. lepromatosis–* and *M. leprae*–specific real-time quantitative PCR (qPCR) on 67 skin lesion biopsies and 25 earlobe slit skin smears (SSS) from 92 multibacillary leprosy patients identified during 2006–2016. The participants were from 11 provinces: Atlántico, Antioquia, Bolívar, Chocó, Cesar, Cundinamarca, Magdalena, Santander, Norte de Santander, Sucre, and Tolima. All samples belonged to the Colombian Institute of Tropical Medicine (Antioquia, Colombia) and were stored in 70% ethanol. Before sample collection, all participants gave written informed consent for future research, and the institutional ethics committee for human research at CES University endorsed such use. We processed samples at the National Hansen’s Disease Program (NHDP) Laboratory (Baton Rouge, LA, USA). We conducted *M. lepromatosis–* and *M. leprae–*specific qPCR on these samples following DNA extraction with DNeasy Kit (QIAGEN, https://www.qiagen.com) and using previously described primers and probes (*4*,*5*). Both these qPCR tests are Clinical Laboratory Improvement Amendments validated and are now used as routine diagnostic tests at the NHDP (*6*).

Of the study participants, 87% were male. Median age was 51.5 years (range 12–84 years). Thirty-seven percent of the participants lived in Santander and 34.8% in Atlantic Coast (Appendix Table 1). qPCRs amplified the repetitive element region specific to *M. lepromatosis* in 5 patients and the repetitive element region specific to *M. leprae* in all samples evaluated. Thus, 5.4% of the patient samples were positive for both *M. leprae* and *M. lepromatosis* and 94.6% (87 patients) were positive for *M. leprae* only (Table). The 5 patients co-infected with *M. lepromatosis* and *M. leprae* resided in geographic areas with a high burden of leprosy: Santander, Atlántico, and Chocó. Four had lepromatous leprosy (LL) and one had dimorphic LL; 1 of the patients had a history of type I leprosy reaction (Appendix Table 2).

**Table:** *Mycobacterium lepromatosis* real-time quantitative PCR results for samples from 92 multibacillary leprosy patients identified during 2006–2016 in Columbia*

Characteristic	No. (%) positive
Biological samples	
Skin biopsies	67 (72.8)
SSS	25 (27.2)
Quantitative PCR	
RLEP positive (*M. leprae*)	87 (94.6)
*M. lepromatosis* and *M. leprae* positive	5 (5.4)

*RLEP, repetitive element in *M. leprae,* SSS, slit skin smears.

Most leprosy-endemic countries do not conduct routine surveillance for *M. lepromatosis,* and so its true distribution and clinical effect are unknown as of 2022. However, this knowledge is crucial for clinical management and to understand the transmission network of leprosy-causing organisms. The earliest known population-based study to analyze the presence of both mycobacteria indicates that *M. lepromatosis* arrived in America with human populations that migrated from Asia through the Bering Strait, in contrast to *M. leprae*, which arrived in America with the settlers and as a result of the slave trade (*7*). To clarify the clinical outcomes of *M. lepromatosis* infection, a study in Mexico associated both mycobacteria with the forms already classified by Ridley and Jopling (*8*). That study found that, of the 55 cases with *M. lepromatosis* as the sole etiologic agent, 34 manifested LL, 13 developed diffuse LL, and the remaining 8 had other forms of leprosy. Fourteen patients carried both mycobacteria and showed all clinical forms (*2*). In contrast, 15% of leprosy patients in Brazil who had *M. lepromatosis* as the sole agent had polar tuberculoid leprosy, none had LL, and patients with infection by both mycobacteria had LL (*7*). The same study evaluated 8 patients in Myanmar and found *M. lepromatosis* in 2 patients, both of whom had LL (*7*).

This study demonstrates presence of *M. lepromatosis* in samples taken by our research group before 2008 when this mycobacterium was first reported (*1*). Therefore, we infer that *M. lepromatosis* has coexisted with *M. leprae* in Colombia for some time. Finally, this report confirms *M. lepromatosis* in Colombia. Genomic surveillance is needed to monitor the infection dynamics of both mycobacteria among leprosy patients and contacts to stop transmission and limit the dire physical, social, economic, and emotional consequences that these organisms cause among susceptible persons.

Appendix Additional information about *M. lepromatosis* as a cause of leprosy, Colombia.
