# Supplementary material for: Mycobacterium lepromatosis as Cause of Leprosy, Colombia
Source: Emerg Infect Dis. 2022 May;28(5):1067–8. doi: 10.3201/eid2805.212015 (PMC9045448; doi:10.3201/eid2805.212015)
Supplement: Appendix — Additional information about M. lepromatosis as a cause of leprosy, Colombia. [file 21-2015-Techapp-s1.pdf]

Article DOI: https://doi.org/10.3201/eid2805.212015

Mycobacterium lepromatosis as Cause of Leprosy, Colombia

Appendix

Appendix Table 1. Demographics and clinical characteristics of *Mycobacterium lepromatosis* and *M. leprae* positive patients.

Patients	n (%)	*M. lepromatosis*– + *M. leprae*–positive samples	Only *M.leprae*–positive samples
Sex			
M	80 (87)	5	75
F	12 (13)	0	12
Median age	51.5 (12-84)		
Year of sampling			
2006–2007	24 (26.1)	1	23
2008–2009	32 (34.8)	3	29
2010–2011	2 (2.2)	1	1
2012–2013	7 (7.6)	-	7
2014-2015	10 (10.9)	-	10
2016	17 (18.5)	-	17
Geographic area			
Santander	34 (37)	3	31
Antioquia	9 (9.8)	0	9
Cundinamarca	8 (8.7)	0	8
Chocó	4 (4.3)	1	3
Atlantic Coast (Bolívar, Sucre, Magdalena, Atlántico)	32 (34.8)	1	31
Other	5 (5.4)	-	5
WHO classification MB	92 (100)	5	87
R&J classification			
BT	8 (8.7)	–	8
BL	10 (10.9)	1	9
LL	74 (80.4)	4	70
Leprosy reactions			
Type I	9 (9.8)	1	8
Type II	17 (18.5)	–	17
Disability grade 2	26 (28.3)	5	21
AFB +	92 (100)	5	87

*AFB, acid fast bacilli; BL, borderline lepromatous leprosy; BT, borderline tuberculoid leprosy; MB, multibacillary; LL, lepromatous leprosy.

Appendix Table 2. Clinical and demographic description of patients infected with both *Mycobacterium leprae* and *M. lepromatosis,* Colombia*

Patient no.	Year of sample	Province	Clinical outcome	Leprosy reaction	Grade disability
1	2007	Atlántico	BL	None	1
2	2008	Santander	LL	None	0
3	2008	Santander	LL	Type I	0
4	2008	Santander	LL	None	0
5	2010	Chocó	LL	None	0

*BL, borderline lepromatosis; LL, lepromatous leprosy
